# A study on the improvement in the ability of endoscopists to diagnose gastric neoplasms using an artificial intelligence system

**DOI:** 10.3389/fmed.2024.1323516

**Published:** 2024-01-29

**Authors:** Bojiang Zhang, Wei Zhang, Hongjuan Yao, Jinggui Qiao, Haimiao Zhang, Ying Song

**Affiliations:** ^1^Department of Gastroenterology, Xi’an Gaoxin Hospital, Xi’an, China; ^2^Clinical Medical College, Xi’an Medical University, Xi’an, China; ^3^College of Nursing and Rehabilitation, Xi’an Medical University, Xi’an, China

**Keywords:** artificial intelligence, AI-assisted gastroscopy, endoscopists, gastric neoplasm, diagnosis

## Abstract

**Background:**

Artificial intelligence-assisted gastroscopy (AIAG) based on deep learning has been validated in various scenarios, but there is a lack of studies regarding diagnosing neoplasms under white light endoscopy. This study explored the potential role of AIAG systems in enhancing the ability of endoscopists to diagnose gastric tumor lesions under white light.

**Methods:**

A total of 251 patients with complete pathological information regarding electronic gastroscopy, biopsy, or ESD surgery in Xi’an Gaoxin Hospital were retrospectively collected and comprised 64 patients with neoplasm lesions (excluding advanced cancer) and 187 patients with non-neoplasm lesions. The diagnosis competence of endoscopists with intermediate experience and experts was compared for gastric neoplasms with or without the assistance of AIAG, which was developed based on ResNet-50.

**Results:**

For the 251 patients with difficult clinical diagnoses included in the study, compared with endoscopists with intermediate experience, AIAG’s diagnostic competence was much higher, with a sensitivity of 79.69% (79.69% vs. 72.50%, *p* = 0.012) and a specificity of 73.26% (73.26% vs. 52.62%, *p* < 0.001). With the help of AIAG, the endoscopists with intermediate experience (<8 years) demonstrated a relatively higher specificity (59.79% vs. 52.62%, *p* < 0.001). Experts (≥8 years) had similar results with or without AI assistance (with AI vs. without AI; sensitivities, 70.31% vs. 67.81%, *p* = 0.358; specificities, 83.85% vs. 85.88%, *p* = 0.116).

**Conclusion:**

With the assistance of artificial intelligence (AI) systems, the ability of endoscopists with intermediate experience to diagnose gastric neoplasms is significantly improved, but AI systems have little effect on experts.

## Introduction

Gastric cancer (GC) is the fifth most common malignant tumor in the world and was the third leading cause of cancer-related deaths worldwide in 2018 (1). The 5 years survival rate for patients with advanced gastric cancer is between 5% and 25%, while the 5 years survival rate for patients with early gastric cancer is as high as 90% (1, 2). Therefore, reducing the misdiagnosis rate of early gastric cancer (EGC) is important for improving the 5 years survival rate and prognosis. However, the diagnosis rate of early gastric cancer in China is less than 10%, and the predicament regarding the diagnosis and treatment of early gastric cancer is severe.

White-light digestive endoscopy (WLE) is the most pivotal way of detecting GC at an early stage (3). In addition, it is recommended that lesions with high-risk features, such as spontaneous bleeding, protrusions, depressions, boundaries, and surfaces, in WLE undergo further observation with magnifying image-enhanced endoscopy (M-IEE) or biopsy sampling (4, 5). Potential high-risk lesions not found with screening endoscopy account for as much as 20% to 40% of the missed diagnoses of EGC (6–8). Therefore, it is vital to improve the capability of WLE in screening high-risk gastric lesions.

Factors that affect the ability to screen for high-risk lesions using WLE mainly include the quality of endoscopic imaging, pre-operative preparation, duration of the gastroscopic examination, coverage rate of the gastroscopic examination sites, and knowledge reserve and experience of endoscopists regarding high-risk lesions. The first four factors mainly involve the quality control of gastroscopy operations, and existing measures can easily achieve homogeneity. However, owing to differences in the training methods, self-learning ability, and clinical operational experience of endoscopists, there are significant differences in their skills when detecting high-risk lesions and identifying endoscopic appearances, and their diagnostic abilities vary greatly.

In recent years, owing to the maturity of algorithms and the efficiency of computing power, artificial intelligence (AI) has made great progress in the field of medicine and plays an indispensable role in medical practice (9). There have been numerous studies using AI technology for the identification of digestive tract lesions on endoscopic images and quality control, mainly focusing on early esophageal cancer identification, esophageal cancer capillary classification, early gastric cancer diagnosis under staining magnification, *Helicobacter pylori* infection identification, gastric cancer differentiation, the monitoring of 26 blind spots in gastroscopic examination, and the evaluation of esophagogastric varices (5, 10–16). Based on previous research, Wu et al. constructed an AI-assisted gastroscopy (AIAG) system (ENDOANGEL-LD) based on ResNet-50 to identify gastric neoplasm lesions under white light and evaluated its effects on the detection rate of gastric neoplasm lesions and the misdiagnosis rate of gastric neoplasm lesions in a randomized controlled serial trial. The study defined gastric neoplasm lesions based on the Vienna classification: mucosal low-grade epithelial tumors (3rd stage), mucosal high-grade epithelial tumors (4th stage), and submucosal invasive cancer (5th stage) (17).

However, there is still insufficient evidence for the assistant efficacy of AIAG in difficult early gastric diagnosis for endoscopists with different levels of experience. This study further used ENDOANGEL-LD (18) to compare the improvement in the ability of endoscopists to identify gastric tumor lesions with AI assistance through retrospective cases that were clinically difficult to diagnose and to demonstrate the actual clinical value of AIAG in the real world.

## Methods

### The AIAG system (ENDOANGEL-LD)

**Figure 1:**
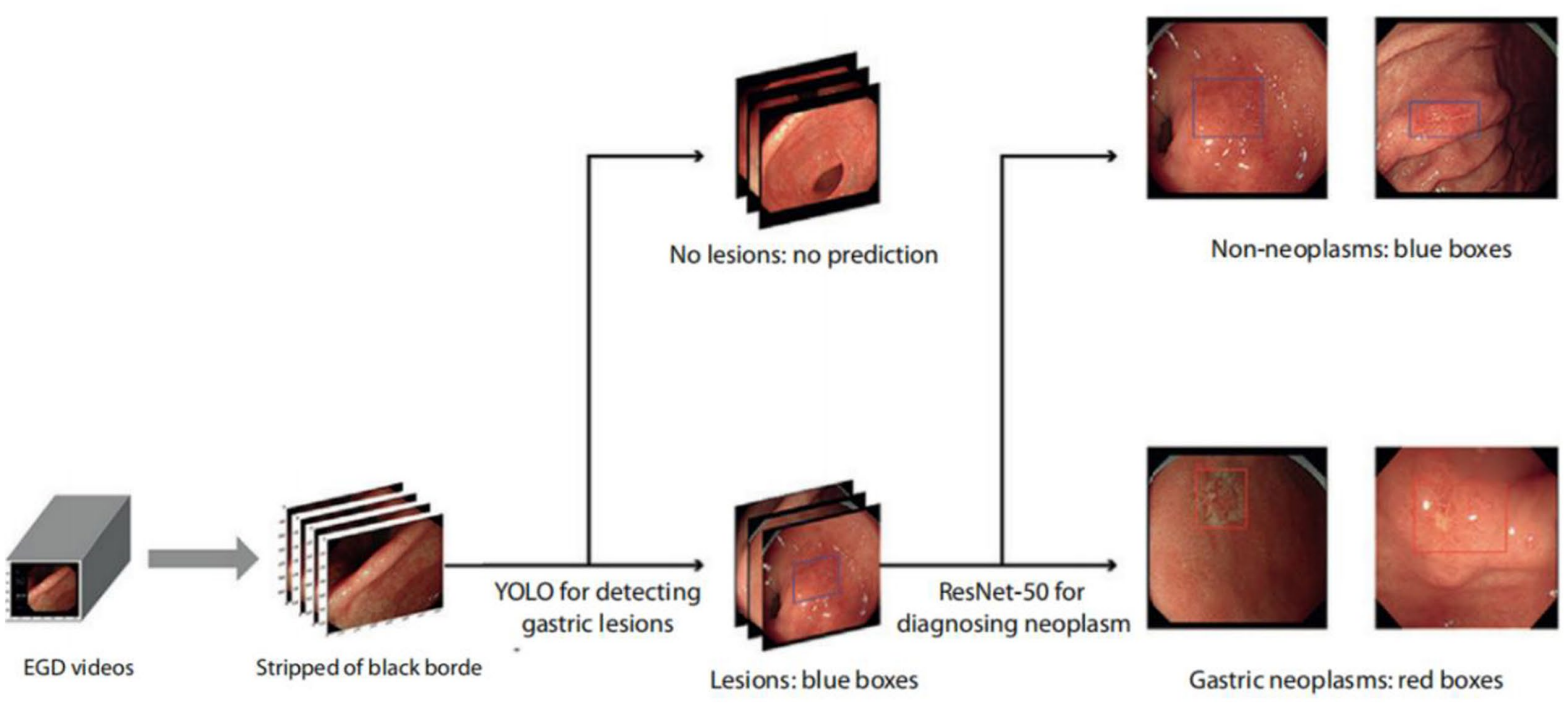
Schematic diagram of the ENDOANGEL-LD system. EGD videos were processed at 15 frames per second, stripped of the black border, and input into deep learning models. Gastric lesions on images were then detected and localized by YOLOv3 and classified as suspicious gastric neoplasms or non-neoplasms on WLI. All lesions were first localized by blue boxes, and those predicted as gastric neoplasms were then focused by red boxes (17). WLI, white-light imaging; AI, artificial intelligence.

In the present study, the training set of the AIAG system (ENDOANGEL-LD) used 9,824 gastric images with labeled lesions, comprising 5,359 images of gastric neoplasm lesions and 4,465 images of non-neoplasm lesions, which were obtained from 12,347 patients who underwent white light gastroscopy examinations at the Renmin Hospital of Wuhan University. As previously reported, in the internal and external validation datasets, the sensitivities were 96.9% and 95.6% for detecting gastric lesions and 92.9% and 91.7% for diagnosing neoplasms, respectively (18). In 2010 prospective consecutive patients, AIAG achieved a sensitivity of 91.8% and specificity of 92.4% for diagnosing neoplasms (17). The schematic diagram of the AIAG system is shown in Figure 1.

### Study design and data collection

This study was conducted at Xi’an Gaoxin Hospital, from June 15th, 2021, to February 9th, 2022. All the initially collected gastroscopy cases underwent preliminary screening and further endoscopic examination, and the risk of lesions was determined based on pathology diagnosis excluding advanced cancer. Ultimately, the study included 251 patients, comprising 64 patients with neoplasm lesions and 187 patients with non-neoplasm lesions. At the same time, comprehensive data of all patients were collected, including baseline information, endoscopic diagnoses, and pathological diagnoses. The images and videos used in this study were generated by two endoscope brands (Olympus CV-290 and Fujifilm VP-7000 from Japan).

**Figure 2:**
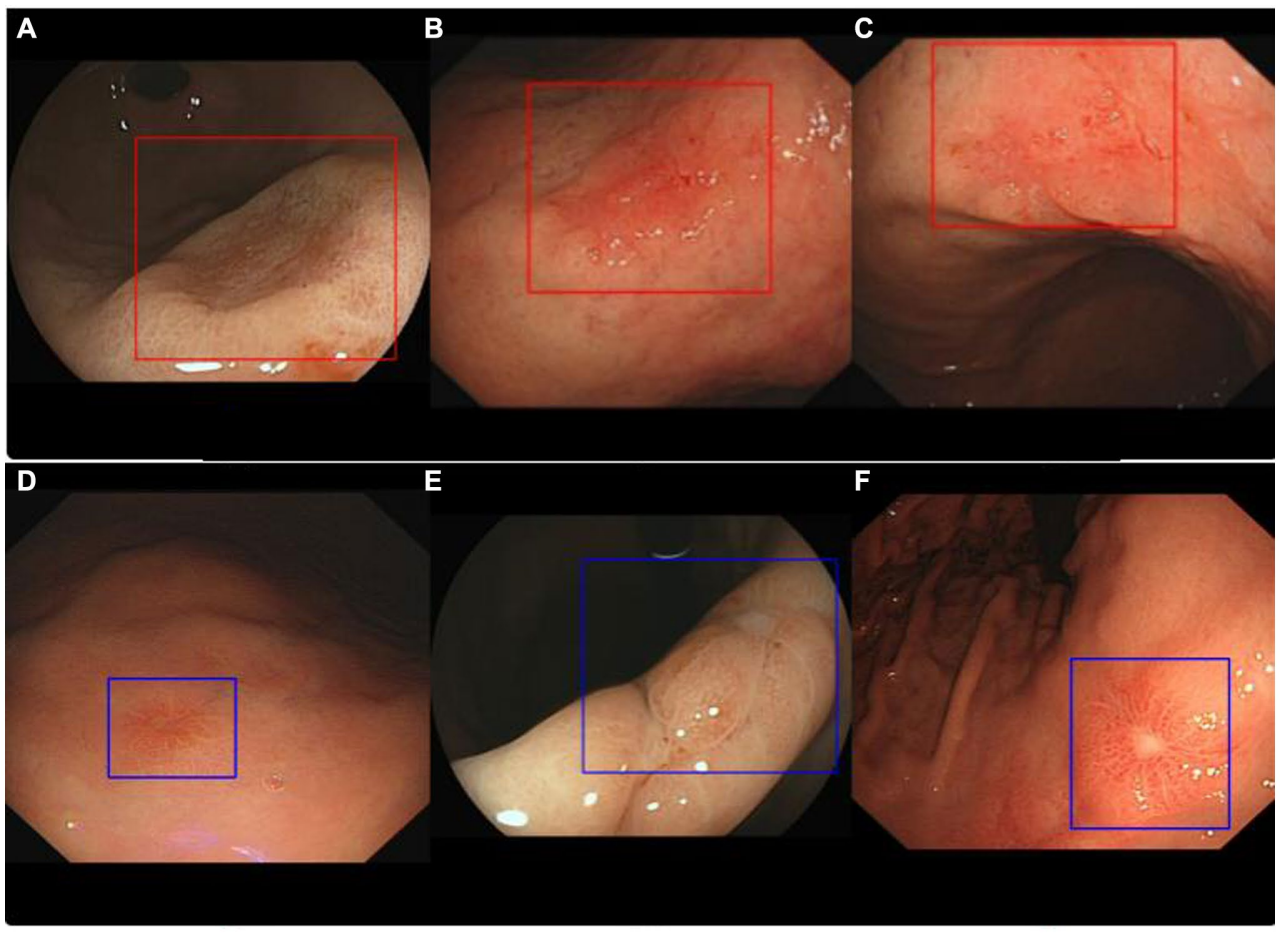
Representative images of ENDOANGEL-LD diagnostic results. Images **(A–C)** show neoplasm lesions that were correctly identified by ENDOANGEL-LD, while images **(D–F)** show non-neoplasms lesions that also were correctly identified.

To ensure that the enrolled cases belonged to clinical diagnoses that were relatively difficult, i.e., lesions without typical traits of gastric neoplasms such as irregular microvascular and irregular microstructure, we arranged for two endoscopic experts (with over 15 years of experience) to conduct data screening and voting. As long as at least one expert thought that the case belonged to a difficult type, it could be included in the study. Difficult cases were defined as a diagnosis with confidence points less than 3 (from 1 to 5 points, for which the confidence level increases with the number of points). Then, the endoscopic images of these patients were input into the AIAG for classification. Figure 2 shows some of the typical images correctly recognized by the AI; neoplasms were diagnosed with red rectangular boxes and non-neoplasm lesions with blue by the AIAG. The study was approved by the Ethics Committee of Xi’an Gaoxin Hospital, and informed consent was not necessary due to the retrospective nature of the study.

Ten endoscopists were recruited in this study and were divided into two groups based on experience, namely, the intermediate group (with less than 8 years of experience in gastroscopy) and the senior group (with more than 8 years of experience in gastroscopy). Each group consisted of five endoscopists. At the same time, they were divided into an independent diagnosis group and an AI-assisted diagnosis group according to the use of AIAG or not.

The pathological diagnoses of gastric neoplasm were based on the category 3 (mucosal low-grade neoplasia), 4 (mucosal high-grade neoplasia), and 5 (submucosal invasion by carcinoma) tumors of the revised Vienna classification, whereas category 1 and 2 tumors were diagnosed as non-neoplasia and category 3 and 4 were diagnosed as gastric neoplasm; the category 5 lesions were excluded.

**Figure 3:**
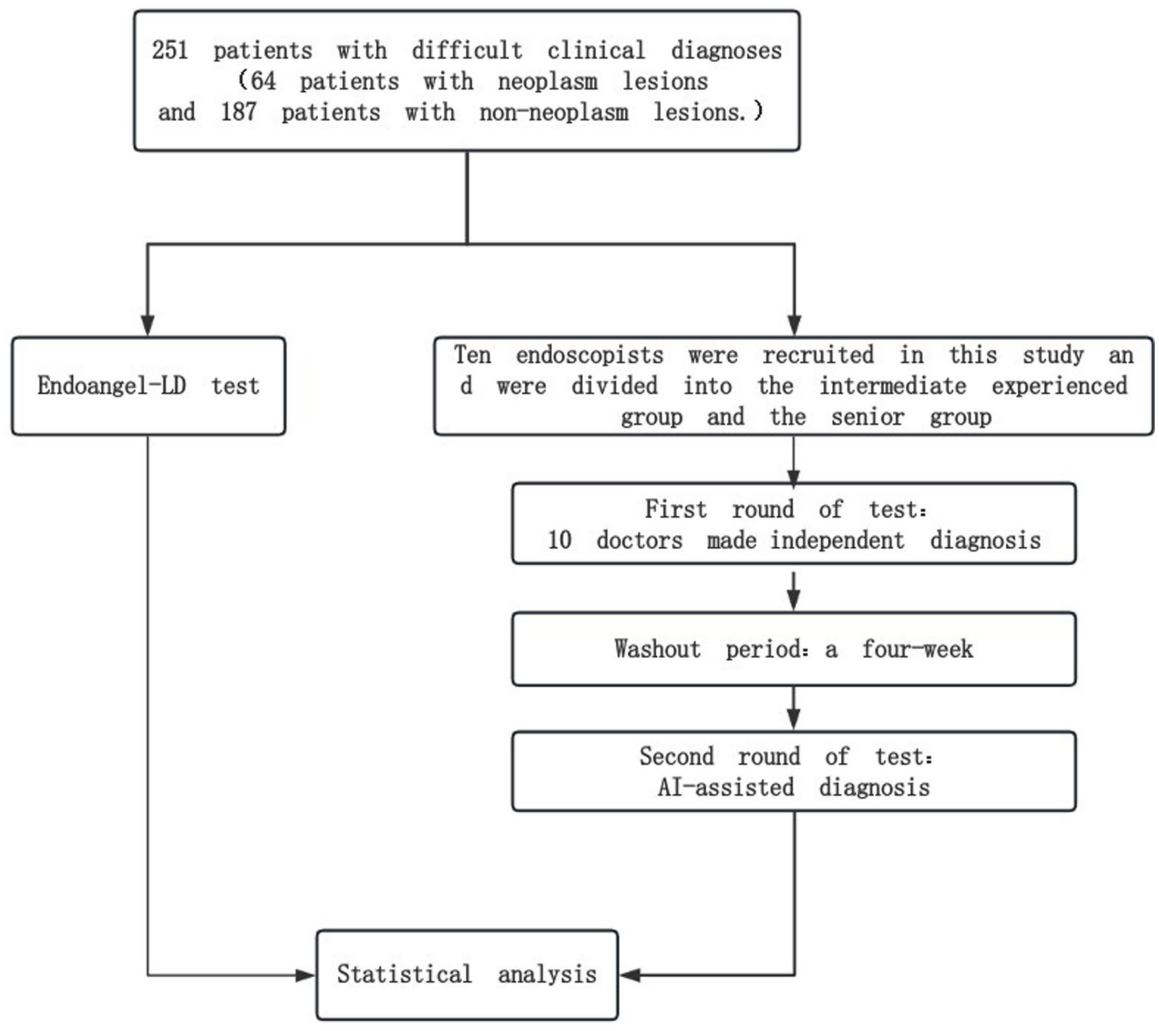
Flow chart of the study.

Then, 10 doctors were randomly and equally assigned to the independent and AI-assisted diagnosis groups; the independent reading group only provided the original endoscopic images of all cases, and the AI-assisted diagnosis group provided the endoscopic images with AIAG recognition results. Both groups gave the diagnosis of neoplastic lesions through an electronic questionnaire (Supplementary material). After the first round of tests, all participants went through a 4 weeks washout period and received the second round of tests. The participants in the first round of independent diagnoses were assigned to the AI-assisted diagnosis group in the second round of tests. The flow chart of the study is shown in Figure 3.

### Evaluation of the AIAG system and comparing it with endoscopists

The performance of the AIAG system was compared with different experienced endoscopists. Taking the pathology diagnosis as the gold standard, the diagnostic sensitivity and specificity were defined as diagnostic sensitivity of the neoplasm = true positive/(true positive + false negative), diagnostic specificity of the neoplasm = true negative/(true negative + false positive).

### Evaluation of the improvement in the ability of endoscopists with the assistance of AI

Taking the pathology diagnosis as the gold standard, the diagnostic ability, including accuracy, sensitivity, and specificity, of different levels of endoscopists was compared with or without the assistance of AIAG.

### Statistical analysis

The quantitative data conforming to the normal distribution are presented as mean ± standard deviation, and the non-normal distribution is presented as the median (interquartile interval). Sensitivity and accuracy were used to evaluate the diagnostic performance of the AIAG system for gastric tumors under white light. The McNemar test was used to compare the diagnostic differences in sensitivity and specificity between endoscopists and AIAG models. *p* < 0.05 was considered as statistically significant. SPSS26.0 was used for statistics.

## Results

### Patient enrollment and baseline information

From June 15th, 2021, to February 9th, 2022, data were retrospectively collected from Xi’an Gaoxin Hospital for patients who received gastroscopy after excluding patients with no pathological histology biopsy, unclear endoscopic images, and endoscopic images with an inappropriate distance and brightness. Then, the endoscopic images of lesions were assessed by two experts and the difficult cases were screened out as described in Methods. Finally, a total of 251 patients were included in the study with a mean age of 51.34 years, of which 176 patients were examined by the Fuji endoscopic system and 75 patients were examined by the Olympus endoscopic system, as shown in Table 1; 64 patients were diagnosed with a neoplasm and 187 were diagnosed with non-neoplasm lesions.

**Table 1:** Baseline information of included patients.

Baseline information		Results
Age (year, mean ± SD)		51.34 ± 12.27
Gender (male/female, %)		(155/96) 61.75
No. of neoplasms		64 (25.50%)
No. of non-neoplasms		187 (74.50%)
Endoscopy brands		
	Number of Olympus	176 (70.12%)
	Number of Fujifilm	75 (29.88%)

### Comparing the competence of the AIAG system with endoscopists

A total of 251 patients included in this study were selected by experts as relatively difficult to clinically diagnose. The diagnostic competence of AIAG was almost equivalent to that of the experts. The diagnostic sensitivity of AIAG was 79.69%, which was higher than that of the experts (79.69% vs. 67.81%), while the diagnostic specificity of AIAG was 73.26%, which was a little lower than that of experts (73.26% vs. 85.88%). Compared with endoscopists with intermediate experience, the diagnostic competence of AIAG was much higher, with a sensitivity of 79.69% and specificity of 73.26%, both of which were higher [79.69% vs. 72.50% (*p* = 0.012) and 73.26% vs. 52.62% (*p* < 0.001), respectively]. More detailed information is shown in Table 2.

**Table 2:** Comparison between the AIAG system and endoscopists.

	AIAG	Experts (95% CI)	Endoscopists with intermediate experience (95% CI)
Sensitivity, %	79.69 (69.83, 89.54)	67.81 (62.35, 72.84)	72.50 (67.20, 77.25)
*p*-value	/	0.221	0.302
Specificity, %	73.26 (66.92, 79.60)	85.88 (83.45, 88.02)	52.62 (49.36, 55.86)
*p*-value	/	0.012	<0.001

### Comparing the competence of the independent diagnosis and AI-assisted diagnosis groups

To further investigate the assistant efficacy of AIAG, we compared the differences in diagnostic sensitivity and specificity of endoscopists with and without the assistance of AIAG. As shown in Table 3, with the help of AIAG, the endoscopists with intermediate experience demonstrated a sensitivity and specificity of 75.00% and 59.79%, respectively, which were both superior to the independent diagnosis group (sensitivity of 72.50% and specificity of 52.62%). There was a statistically significant difference in specificity (*p* < 0.001) but no statistically significant difference in sensitivity (*p* = 0.4158). The sensitivity and specificity of the expert’s AI-assisted group were 70.31% and 83.85%, respectively, which were similar to the sensitivity of 67.81% and specificity of 85.88% of the group without AI assistance. There was no statistically significant difference in sensitivity (*p* = 0.358) and specificity (*p* = 0.116).

**Table 3:** Comparing the competence of the independent diagnosis group and the AI-assisted diagnosis group.

	Experts (95% CI)		Endoscopists with intermediate experience (95% CI)	
	Without AIAG	With AIAG	Without AIAG	With AIAG
Sensitivity, %	67.81 (62.35, 72.84)	70.31 (64.93, 75.20)	72.50 (67.20, 77.25)	75.00 (69.81, 79.57)
*p*-value	0.358		0.4158	
Specificity, %	85.88 (83.45, 88.02)	83.85 (81.30, 86.12)	52.62 (49.36, 55.86)	59.79 (56.56, 62.94)
*p*-value	0.116		<0.001	

## Discussion

In the present study, we retrospectively collected gastroscopy images of patients who were difficult to diagnose and conducted a cross-reading test to investigate the diagnosis competence and assistant competence of AIAG. AIAG demonstrated a similar diagnostic competence to endoscopists with intermediate experience and could effectively improve the specificity of their gastric neoplasm diagnosis.

Deep learning has emerged as a transformative force in various medical applications, and its integration into the field of endoscopy for gastric neoplasm diagnosis represents a significant stride toward more accurate and efficient medical diagnostics (19). Gastric neoplasms, including tumors and abnormal dysplasia in the stomach, pose a considerable health concern, and early detection is crucial for effective treatment. Deep learning algorithms excel at learning patterns and features from vast amounts of data. In the context of endoscopy for gastric neoplasm diagnosis, these algorithms are trained on extensive datasets containing images and videos of normal and abnormal gastric lesions (5, 11, 19).

Recent advancements underscore substantial differences in the skills of endoscopists, particularly in detecting high-risk lesions and discerning endoscopic appearances. The wide variability in diagnostic abilities among endoscopists is attributed to differences in training methods, self-learning aptitude, and clinical operational experience (18, 19). Typically, endoscopists diagnose gastric tumors based on color, shape, blood vessels, gland duct, and lesion site boundaries. By contrast, AI systems employ a deep convolutional neural network model (DCNN) to learn from a comprehensive dataset of previously confirmed lesions during training and validation. The AI system then applies this knowledge to diagnose actual clinical test images. By comparing the test set with images from the training/validation set, the AI system accumulates a wealth of macroscopic and microscopic lesion details, including color, morphology, blood vessels, gland vessels, and boundaries (11). This continuous accumulation during the training/validation set not only results in a large repository of lesion images but also leads to a higher diagnostic accuracy than that of less experienced endoscopists. Likewise, experienced endoscopists, due to their extensive work tenure and accumulated lesion images, are expected to exhibit a higher diagnostic accuracy than their less experienced counterparts.

This study affirmed a notable discrepancy in diagnostic proficiency for gastric neoplasm diagnosis between experts and endoscopists with intermediate experience. Expert endoscopists demonstrated significantly superior diagnostic competence than their intermediate counterparts. Furthermore, the study ascertained that the diagnostic capacity of AIAG aligned closely with that of experts. Subsequently, through a cross-reading trial, the research delved into the nuanced exploration of AIAG’s impact on improving gastric neoplasm diagnostic competence. The findings revealed a substantial role played by AI in enhancing the diagnostic abilities of endoscopists with intermediate experience, showing improvements in specificity. This empirical evidence substantiated the potential of AI as a valuable adjunct tool in bolstering the diagnostic acumen of healthcare practitioners at intermediate stages of their professional development.

The low competence of endoscopists with intermediate experience at diagnosing gastric neoplasm and the low sensitivity of experts may account for two potential reasons. First, only limited gastric neoplasm cases were included in the dataset due to the nature of the low incidence rate and low endoscopic detection rate of gastric neoplasm lesions, which contributed to a relatively unbalanced distribution of gastric neoplasms and normal lesions in the dataset. As a result, any misdiagnosis of gastric neoplasm cases could lead to an obvious decrease in sensitivity for gastric neoplasm detection. Second, the included gastric neoplasm lesions were evaluated as “difficult” cases that did not demonstrate the typical traits of neoplasms, such as spontaneous bleeding, protrusions, depressions, boundaries, and surfaces, which contributed to the lack of confidence in diagnosing gastric neoplasms, resulting in the low sensitivity of experts and low competence of endoscopists with intermediate experience.

There were some limitations in the present study. First, this study was a single-center retrospective study, and its results still need to be confirmed by a multi-center large-sample prospective randomized controlled study. Second, AIAG showed a lower performance in gastric neoplasm diagnosis than in the previous report. The low performance may be attributed to the difficult cases of gastric neoplasm lesions adopted in the present study. More samples for atypical gastric neoplasm lesions should be collected to optimize the competence of AIAG in the future.

In conclusion, this study verified that the AI system had a comparable diagnostic capability for diagnosing gastric neoplasms with experts in cases with difficult clinical diagnoses. In addition, with the assistance of AI, the diagnostic level of endoscopists with intermediate experience will be improved, especially in specificity. These outcomes contribute to the burgeoning academic discourse surrounding the collaborative synergy between human expertise and AI technology, offering valuable insights into the ongoing refinement of diagnostic practices for gastric neoplasms.

## Data availability statement

The original contributions presented in the study are included in the article/Supplementary material, further inquiries can be directed to the corresponding author.

## Ethics statement

Ethical approval was not required for the study involving humans in accordance with the local legislation and institutional requirements. Written informed consent to participate in this study was not required from the participants or the participants’ legal guardians/next of kin in accordance with the national legislation and the institutional requirements.

## Author contributions

BZ: Conceptualization, Data curation, Formal analysis, Investigation, Methodology, Writing – original draft. WZ: Methodology, Project administration, Supervision, Validation, Writing – review & editing. HY: Investigation, Methodology, Resources, Writing – review & editing. JQ: Conceptualization, Data curation, Formal analysis, Investigation, Methodology, Project administration, Writing – review & editing. HZ: Investigation, Methodology, Resources, Writing – review & editing. YS: Conceptualization, Data curation, Formal analysis, Investigation, Methodology, Project administration, Supervision, Validation, Visualization, Writing – original draft, Writing – review & editing.

## References

[1] Banks M Graham D Jansen M Gotoda T Coda S di Pietro M. British Society of Gastroenterology guidelines on the diagnosis and management of patients at risk of gastric adenocarcinoma. Gut. (2019) 68:1545–75. doi: 10.1136/gutjnl-2018-318126, PMID: 31278206 PMC6709778

[2] Sáftoiu A Hassan C Areia M Bhutani MS Bisschops R Bories E. Role of gastrointestinal endoscopy in the screening of digestive tract cancers in Europe: European Society of Gastrointestinal Endoscopy (ESGE) position statement. Endoscopy. (2020) 52:293–304. doi: 10.1055/a-1104-5245, PMID: 32052404

[3] Cortes C Mehryar M Afshin R. (2009) *L*_2_ regularization for learning kernels. UAI ’09: Proceedings of the Twenty-Fifth Conference on Uncertainty in Artificial Intelligence. 109–116.

[4] Tanner MA Wong WH. The calculation of posterior distributions by data augmentation. J Am Stat Assoc. (1987) 82:528–40. doi: 10.1080/01621459.1987.10478458

[5] Wu L Zhang J Zhou W An P Shen L Liu J. Randomised controlled trial of WISENSE, a real-time quality improving system for monitoring blind spots during esophagogastroduodenoscopy. Gut. (2019) 68:2161–9. doi: 10.1136/gutjnl-2018-317366, PMID: 30858305 PMC6872441

[6] Evans JA Chandrasekhara V Chathadi KV Decker GA Early DS Fisher DA. The role of endoscopy in the management of premalignant and malignant conditions of the stomach. Gastrointest Endosc. (2015) 82:1–8. doi: 10.1016/j.gie.2015.03.1967, PMID: 25935705

[7] Rutter MD Senore C Bisschops R Domagk D Valori R Kaminski MF. The European Society of Gastrointestinal endoscopy quality improvement initiative: developing performance measures. Endoscopy. (2016) 48:81–9. doi: 10.1055/s-0035-1569580, PMID: 26662057

[8] Kaise M. Advanced endoscopic imaging for early gastric cancer. Best Pract Res Clin Gastroenterol. (2015) 29:575–87. doi: 10.1016/j.bpg.2015.05.010 26381303

[9] He J Baxter SL Xu J Xu J Zhou X Zhang K. The practical implementation of artificial intelligence technologies in medicine. Nat Med. (2019) 25:30–6. doi: 10.1038/s41591-018-0307-0, PMID: 30617336 PMC6995276

[10] Wu L Zhou W Wan X Zhang J Shen L Hu S. A deep neural network improves endoscopic detection of early gastric cancer without blind spots. Endoscopy. (2019) 51:522–31. doi: 10.1055/a-0855-3532, PMID: 30861533

[11] Wu L He X Liu M Xie H An P Zhang J. Evaluation of the effects of an artificial intelligence system on endoscopy quality and preliminary testing of its performance in detecting early gastric cancer: a randomized controlled trial. Endoscopy. (2021) 53:1199–207. doi: 10.1055/a-1350-5583, PMID: 33429441

[12] Zhao YY Xue DX Wang YL Zhang R Sun B Cai YP. Computer-assisted diagnosis of early esophageal squamous cell carcinoma using narrow-band imaging magnifying endoscopy. Endoscopy. (2019) 51:333–41. doi: 10.1055/a-0756-8754, PMID: 30469155

[13] Zhang M Zhu C Wang Y Kong Z Hua Y Zhang W. Differential diagnosis for esophageal protruded lesions using a deep convolution neural network in endoscopic images. Gastrointest Endosc. (2021) 93:1261–1272.e2. doi: 10.1016/j.gie.2020.10.005, PMID: 33065026

[14] Tang D Wang L Ling T Lv Y Ni M Zhan Q. Development and validation of a real-time artificial intelligence-assisted system for detecting early gastric cancer: a multicentre retrospective diagnostic study. EBioMedicine. (2020) 62:103146. doi: 10.1016/j.ebiom.2020.103146, PMID: 33254026 PMC7708824

[15] Zheng W Zhang X Kim JJ Zhu X Ye G Ye B. High accuracy of convolutional neural network for evaluation of *Helicobacter pylori* infection based on endoscopic images: preliminary experience. Clin Transl Gastroenterol. (2019) 10:e00109. doi: 10.14309/ctg.0000000000000109, PMID: 31833862 PMC6970551

[16] Chen M Wang J Xiao Y Wu L Hu S Chen S. Automated and real-time validation of gastroesophageal varices under esophagogastroduodenoscopy using a deep convolutional neural network: a multicenter retrospective study (with video). Gastrointest Endosc. (2021) 93:422–432.e3. doi: 10.1016/j.gie.2020.06.058, PMID: 32598959

[17] Wu L Shang R Sharma P Zhou W Liu J Yao L. Effect of a deep learning-based system on the miss rate of gastric neoplasms during upper gastrointestinal endoscopy: a single-centre, tandem, randomised controlled trial. Lancet Gastroenterol Hepatol. (2021) 6:700–8. doi: 10.1016/S2468-1253(21)00216-8, PMID: 34297944

[18] Wu L Xu M Jiang X He X Zhang H Ai Y. Real-time artificial intelligence for detecting focal lesions and diagnosing neoplasms of the stomach by white-light endoscopy (with videos). Gastrointest Endosc. (2022) 95:269–280.e6. doi: 10.1016/j.gie.2021.09.017, PMID: 34547254

[19] Murakami D Yamato M Amano Y Nishino T Arai M. Variation in the rate of detection of minute and small early gastric cancers at diagnostic endoscopy may reflect the performance of individual endoscopists. BMJ Open Gastroenterol. (2023) 10:e001143. doi: 10.1136/bmjgast-2023-001143, PMID: 37407230 PMC10335432

